# Screening cell mechanotype by parallel microfiltration

**DOI:** 10.1038/srep17595

**Published:** 2015-12-02

**Authors:** Dongping Qi, Navjot Kaur Gill, Chintda Santiskulvong, Joshua Sifuentes, Oliver Dorigo, Jianyu Rao, Barbie Taylor-Harding, W. Ruprecht Wiedemeyer, Amy C. Rowat

**Affiliations:** 1 Department of Integrative Biology and Physiology, University of California, Los Angeles, USA; 2 Department of Pathology and Laboratory Medicine, David Geffen School of Medicine, University of California, Los Angeles, USA; 3 Department of Obstetrics and Gynecology, Division Gynecologic Oncology, Stanford Cancer Institute, Stanford University, USA; 4 Women’s Cancer Program, Samuel Oschin Comprehensive Cancer Institute, Cedars-Sinai Medical Center, Los Angeles, USA; 5 Department of Obstetrics and Gynecology, David Geffen School of Medicine, University of California Los Angeles, USA

## Abstract

Cell mechanical phenotype or ‘mechanotype’ is emerging as a valuable label-free biomarker. For example, marked changes in the viscoelastic characteristics of cells occur during malignant transformation and cancer progression. Here we describe a simple and scalable technique to measure cell mechanotype: this parallel microfiltration assay enables multiple samples to be simultaneously measured by driving cell suspensions through porous membranes. To validate the method, we compare the filtration of untransformed and HRas^V12^-transformed murine ovary cells and find significantly increased deformability of the transformed cells. Inducing epithelial-to-mesenchymal transition (EMT) in human ovarian cancer cells by overexpression of key transcription factors (Snail, Slug, Zeb1) or by acquiring drug resistance produces a similar increase in deformability. Mechanistically, we show that EMT-mediated changes in epithelial (loss of E-Cadherin) and mesenchymal markers (vimentin induction) correlate with altered mechanotype. Our results demonstrate a method to screen cell mechanotype that has potential for broader clinical application.

Cells are viscoelastic materials whose mechanotype is altered in diseases from malaria to cancer^1^,^2^. For example, malignant cells across different types of cancers are consistently 2–5× softer than benign cells both *in vitro* and *in situ*^3^,^4^,^5^,^6^. Cell mechanotype also grades metastatic potential: highly invasive human ovarian carcinoma cells are up to 5× softer than less invasive cells^3^,^4^. Mechanotyping of patient samples shows potential for clinical diagnoses of cancer^7^. Moreover, the compliance of cancer cells is altered by chemotherapy drugs. For example, leukemia cells exhibit a ~10 to 100-fold increase in elastic modulus after being treated with dexamethasone and daunorubicin^8^. While cell mechanotype has potential as a biomarker in cancer diagnosis and for identifying effective drug treatments, to efficiently screen cell mechanotype for fundamental research and clinical applications requires a simple and scalable method.

Various techniques provide quantitative insight into the viscoelastic behavior of cells including micropipette aspiration^9^,^10^, atomic force microscopy^6^,^11^, and cantilever compression^12^,^13^. These methods enable detailed characterization of the force-deformation response of typically <10^2^ individual cells, which limit the number of independent samples that can be probed within a reasonable timescale. An alternative way to measure cell deformability is to filter cells through membranes with micron-scale pores^14^,^15^,^16^; however, these measurements are performed sequentially, which limits scale-up. More recently, microfluidic methods enable more efficient measurements of cell mechanotype: real-time deformability cytometry probes the deformation of single cells at ~100 cells/s^17^ and requires over 1 hour to obtain data on a single sample from the initial state of cells in culture. Cells can also be deformed by the shear and compressive stresses generated as cells flow through micron-scale constrictions^18^,^19^,^20^, or through opposing fluid streams^21^; while these methods enable measurements at rates of up to ~2,000 cells/sec, the total measurement time for a single sample is approximately 1.5 hours as high-speed imaging and intensive computational analysis is required; this also challenges the measurement of different samples in parallel. If we could rapidly assess the deformability of multiple samples in a single measurement, we could harness the intrinsic mechanotype of cells for practical applications.

Here we describe a parallel microfiltration (PMF) method that enables simultaneous measurements of cell mechanotype across multiple samples. We use uniform air pressure to drive cell suspensions through porous membranes; the relative deformability of a cell sample is quantified by the fraction of sample retained above the membrane. Herein we describe PMF design principles and operation parameters. Based on our experimental results and theoretical considerations, we develop a simple model that provides a physical explanation of PMF and allows us to relate our experimental data to cell deformability. We validate the method by mechanotyping a variety of cancer cell types, including epithelial and mesenchymal-type cells, as well as cells treated with chemotherapy drugs. We focus on human promyelocytic leukemia (HL-60) and ovarian cancer cells, as the mechanotype of these cells has been characterized using other complementary techniques^3^,^4^,^11^,^22^,^23^.

## Results

### Parallel microfiltration concept

**Figure 1:**
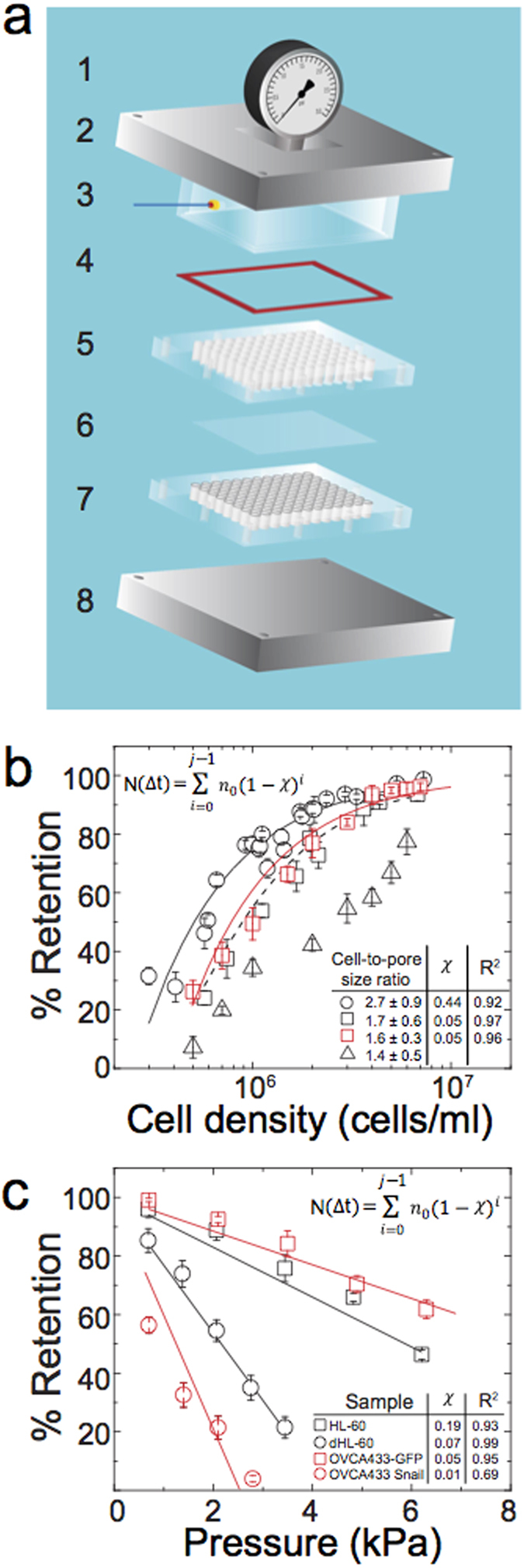
Overview of the parallel microfiltration (PMF) platform. (**a**) Schematic illustration of the parallel filtration platform. 1: Pressure gauge; 2: Aluminum plate for assembling; 3: Pressure chamber. An air pressure source (Supplementary Fig. 10) connects via the blue tube; 4: Silicone sealing pad; 5: 96-well loading plate; 6: Porous membrane; 7: 96-well bottom plate with O-rings (black); 8: Aluminum plate for assembling. (**b**) Dependence of HL-60 cell filtration on cell density through membranes of 5, 8, and 10 μm pores. Black circles: 5 μm pore membrane, 3.4 kPa applied for 20 s; black squares: 8 μm pore membrane, 0.7 kPa applied for 20 s; black triangles: 10 μm pore membrane, 0.7 kPa applied for 20 s. Red squares show filtration of ovarian cancer cells (OVCA433-Snail) through 10 μm pore membrane, 2.1 kPa for 50 s. Lines represent model fitting obtained using the least squares method: solid lines for fitting data shown by black circles and red squares; fitting of the black squares is denoted with a dashed line. (**c**) Pressure dependence of HL-60 control (black squares) versus ATRA-treated (dHL-60, black circles) cell filtration with 5 μm pore membrane for 20 s. Filtration of ovarian cancer cells OVCA433-GFP control (red squares) versus OVCA433-Snail (red circles) with 10 μm pore membrane for 50 s. For (**b**) and (**c**), filtration and modeling parameters are shown in the insets. Solid lines represent model fitting obtained using the least squares method. Each data point represents mean ± S.D.

The essential components of the PMF device are shown in Fig. 1a. Polycarbonate membranes are sandwiched between two custom-fabricated 96-well plates; using membranes with varying pore sizes can enable filtration through multiple pore sizes in a single run. We place cell suspensions in the top wells, and apply a uniform pressure gradient across the membrane for a defined period of time. To quantify the filtration of each individual cell sample, we measure the fraction of the initial mass of cell suspension that is retained in the top well, which is the percentage (%) retention; equivalently, the number of retained cells can also be measured (Supplementary Fig. 1).

### Modeling membrane filtration

To understand the essential operation parameters and physical mechanism underlying PMF, we consider fluid flow through porous media, as described by Darcy’s Law, 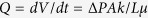, where *Q* is the flow rate; *V*, the flow volume; *t*, the time; ∆*P*, the pressure applied to drive the cell suspension to flow through the micron-scale pores; *A*, the cross-sectional flow area; *L*, the membrane thickness; *μ*, the dynamic viscosity of the cell medium; and *k*, the membrane permeability. When a cell is larger than a pore, it is subject to external stresses that cause it to deform. Whether or not a cell occludes a pore depends on the driving pressure, cell and pore sizes, surface properties, and the cell’s intrinsic mechanical properties^9^,^24^. In each experiment, we maintain constant driving pressure and consistent cell-to-pore size ratios across different cell samples (Supplementary Fig. 2). Previous observations show that the viscoelastic properties of cells, rather than surface interactions, dominate their deformation into a micron-scale pore^25^; we also minimize surface interactions by passivating the wells prior to filtration (**Methods**). We cannot exclude that altered cell surface properties contribute to cell filtration behavior, since friction forces between the cell and pore depend on both cell surface properties^25^ and deformability^26^. Yet cell deformability plays a central role in % retention measurements: we observe a major decrease in % retention for cells whose F-actin structures are disrupted by treatment with cytochalasin D (Supplementary Fig. 3). On the other hand, stabilization of F-actin with colchicine treatment results in increased % retention (Supplementary Fig. 3a). These results are consistent with previous reports of how pharmacologic and genetic perturbations of cytoskeletal or nuclear structures impact the ability of cells to deform through micron-scale pores; these perturbations primarily alter cell deformability, rather than cell surface properties^18^,^25^. Taken together, these results substantiate that the fraction of cells that occlude the pores at a set pressure depends on differences in cell mechanical properties^8^,^19^ and dictates the total volumetric flow: a larger fraction of occluded pores results in less flow through the membrane, and consequently fewer cells will transit through the pores.

To model the filtration process, we consider the time-dependence of membrane occlusion. In a given time window, ∆*t*, a defined number of cells, *N*, that arrive at the membrane and transit the pores is determined by


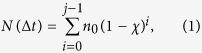


where *n*_*0*_ is the total number of pores in the filtration area, *χ* is the fraction of cells that occlude the pores, and *j* is the total number of iterations. We fit this simple model to the measured filtration data by adjusting only one parameter, *χ,* which reflects cell mechanotype. A lower *χ* value indicates that a smaller fraction of cells occlude the pores; this is consistent with a sample of softer cells, the majority of which readily deform through the micron-scale constrictions in response to applied pressure. By contrast, a higher *χ* value indicates that a larger fraction of cells occlude the pores on the experimental timescale, reflecting a sample of cells that are on average more resistant to deforming through pores of a particular size at a given pressure^18^,^19^,^24^.

### Optimizing sample cell density

The cell density is critical for filtration: with a low cell density, the number of pores exceeds the number of cells, and there is negligible change in the cell suspension flow rate. By contrast, if the cell density is too high, pores are rapidly occluded; similar jamming phenomenon is observed for colloidal suspensions^27^,^28^,^29^. To establish a suitable cell density for filtration, we determine the % retention of HL-60 and ovarian cancer cell samples across a range of densities from ~10^5^ to 10^7^cells/ml. With increasing cell density we observe that the percentage of sample retained increases monotonically (Fig. 1b). One possible origin of this increased retention could be an increased viscosity of the cell suspension due to the higher cell density. However, according to Einstein’s equation to describe the effect of particle volume fraction, *ϕ,* on viscosity, *η* = *η*_*s*_(1+2.5*ϕ*), where *η*_*s*_ is the solvent/medium viscosity. By Darcy’s law, the increase in viscosity between cell samples with concentrations of 10^5^ versus 10^7^ cells/ml would result in less than a 2% variation in suspension flow rate. Thus, the observed ~3-fold increase in % retention cannot be attributed to the altered viscosity of the cell suspension but rather the progressive occlusion of pores.

To evaluate how well our model describes the data, we obtain the best fit of Equation 1 with *χ* as the sole fitting parameter (Fig. 1b,c). For filtration through 5 μm pores, *χ* = 0.44, whereas *χ* = 0.05 for 8 μm pores, reflecting that a smaller fraction of cells occlude the larger pores. Importantly, these results guide us to choose an initial density of less than 10^6^ cells/ml, which equates to a cell-to-pore number ratio of ~10 and ensures measurable filtration in the regime below which % retention saturates with increasing cell density.

### Establishing membrane pore size and filtration pressure

When setting up PMF to assay a particular cell type, both pore size and applied pressure must be selected to optimize the dynamic range of filtration. The cell-to-pore size ratio is typically ~1.2 – 3.1 (Supplementary Fig. 2). The optimal driving pressure depends on the fluidic resistance of the membrane, which is determined by its physical characteristics, such as the size and density of pores, as well as the physical properties of the cells. The applied pressure should be large enough to ensure that cells can deform and transit across the membrane, yet not so excessive that all cells transit through, precluding any measurement of cell mechanotype. To validate PMF, we use HL-60 cells, whose mechanical properties are well characterized^11^,^30^. We establish the driving pressure for filtration through 5 μm pore membranes by performing a pressure sweep from 0.7 to 6.2 kPa. Across this pressure range, we observe a linear dependence of % retention on pressure (Fig. 1c). We then simultaneously filter HL-60 cells and neutrophil-type HL-60 (dHL-60) cells that are differentiated using all-trans retinoic acid (ATRA). These two cell types have similar sizes (Supplementary Fig. 2) but distinct mechanical properties^11^,^30^. While the pressure response is linear for both cell types, there is a marked difference in slope (Fig. 1c), reflecting the difference in cell mechanotype. Fitting our model to this data reveals a larger *χ* parameter for HL-60 cells (*χ* = 0.19, R^2^ = 0.93) than dHL-60 cells (*χ* = 0.07, R^2^ = 0.99), indicating that dHL-60 cells are softer than HL-60 cells. Our results are consistent with previous findings that dHL-60 cells are 2-fold more compliant than HL-60 cells^30^, and have a lower Young’s modulus, E_dHL-60_ ~156 Pa versus E_HL-60_ cells ~855 Pa^11^. We also analyze the pair of human ovarian cancer cells, OVCA433-GFP (control) and OVCA433 that overexpresses *SNAI1*=Snail (OVCA433-Snail) that has undergone epithelial-to-mesenchymal transition (EMT). Previous studies indicate that cancer cell mechanotype is altered with EMT^22^,^23^,^31^,^32^. We observe that the mesenchymal-like OVCA433-Snail cells are softer than the OVCA433-GFP control cells, as shown by the greater reduction in % retention with increasing pressure; fitting our model to this data reveals a larger *χ* parameter for the OVCA433-GFP cells (*χ* = 0.05, R^2^ = 0.95) compared to the OVCA433-Snail cells (*χ* = 0.01, R^2^ = 0.69).

### Predicting epithelial versus mesenchymal-type cells based on mechanotype

**Figure 2:**
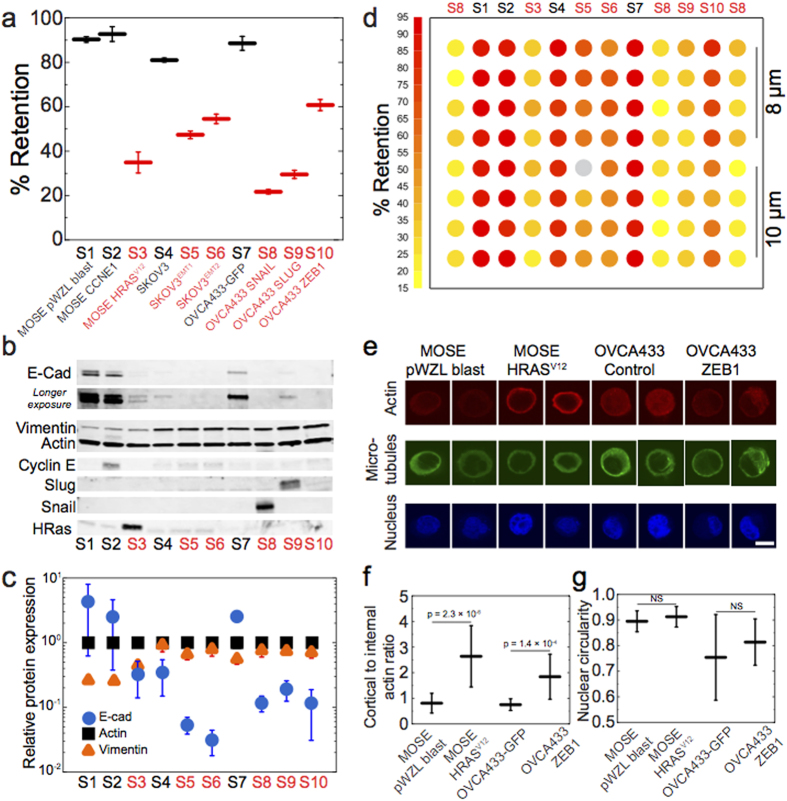
Identifying epithelial versus mesenchymal cells by mechanotype. (**a**) Mouse ovarian surface epithelial (MOSE) and human ovarian cancer cells (SKOV3 and OVCA433) are modified to generate a panel of cells that includes epithelial- (black) and mesenchymal-like (red) cell types. Percentage retention of a panel of mouse and human cell lines after filtration with 10 μm pore membrane at 2.1 kPa for 50 s. Each data point represents mean ± S.D. The modified cells show reduced % retention as compared to the non-modified control: Snail: 22 ± 4%, Slug: 33 ± 4%, and Zeb1: 54 ± 5% versus control OVCA433-GFP: 91 ± 2%. SKOV3^EMT1^: 44 ± 4% and SKOV3^EMT2^: 52 ± 3% while the control SKOV3 cells: 84 ± 3%. Collectively the % retention of all epithelial-type cells is higher than cells with mesenchymal phenotype: 88 ± 5% vs. 41 ± 14%, p = 1.8 × 10^*−*15^. (**b**) Western blot characterization of the cell panel probing for protein biomarkers of epithelial- and mesenchymal-type cells such as E-cadherin (ECad) and vimentin. (**c**) Quantification of E-cadherin and vimentin protein levels normalized to the loading control, actin. Data points represent mean ± S.D. (**d**) Heat map shows % retention for the EMT panel for both 8 and 10 μm membranes. The 10 μm pore size shows reduced variability compared to the 8 μm pore size; therefore we conduct our screens using 10 μm membranes. (**e**) Confocal images of immunolabeled cells. Nuclei are stained with DRAQ5. Two representative images are shown for each cell type. Scale, 10 μm. (**f**) Cortical to internal actin ratio of four representative cell lines from the panel. (**g**) Nuclear circularity.

To test the utility of PMF in screening cancer cell mechanotype, we establish a pore size of 10 μm and driving pressure of 2.1  kPa (Fig. 1c) for filtration of representative epithelial and mesenchymal-type ovarian cancer cells (Fig. 2d). We first validate how transformation of murine ovarian surface epithelial cells (MOSE) by the *HRAS*^*V12*^ oncogene^33^ impacts cell filtration. The mock-transformed (pWZL) MOSE cells show 90 ± 1% retention, whereas the HRas^V12^-expressing MOSE cells show 32 ± 3% retention, indicating the transformed cells are more deformable (Fig. 2a). The MOSE-HRas^V12^ cells also show reduced E-cadherin and elevated vimentin levels, which is consistent with mesenchymal phenotype (Fig. 2b). Another hallmark of mesenchymal-type cells is their greater propensity for colony formation in soft agar compared to epithelial-type cells^34^. Indeed, the MOSE-HRas^V12^ cells form a larger number of colonies compared to the mock-transformed control (Supplementary Fig. 4). We also tested MOSE cells engineered to overexpress cyclin E1, which is encoded by *CCNE1,* a less potent oncogene in this system. These cells express similar levels of E-Cadherin as the MOSE control cells and form only a small number of colonies in soft agar (Supplementary Fig. 4). Consistent with their epithelial phenotype, MOSE-Cyclin E1 cells exhibit similar retention to the MOSE control cells (Fig. 2a).

HRAS^V12^-mediated transformation is accompanied by EMT (Fig. 2), which has critical implications in cancer: cells with mesenchymal phenotype exhibit enhanced motility and increased propensity to detach from the primary tumor^35^. While key changes in protein expression, such as the reduction in E-Cadherin and increase in vimentin during EMT are well-studied^36^,^37^, the changes that occur in mechanotype are not fully understood. Therefore, we next ask if EMT itself leads to increased deformability. To address this question, we probe two individual clones of the human ovarian cancer cell line SKOV3 that underwent EMT, acquiring the mesenchymal phenotype in the process of becoming drug-resistant. Both SKOV3^EMT1^ and SKOV3^EMT2^ clones exhibit a reduction in E-cadherin expression (Fig. 2b); they also show a reduced % retention compared to the control SKOV3 cells (Fig. 2a). These results suggest that the process of EMT is accompanied by reduced stiffness.

To test this hypothesis directly, we overexpress single genes that are master regulators of EMT (*SNAI1* = Snail, *SNAI2* = Slug, *ZEB1* = Zeb1). Here, we use a human ovarian cancer cell line, OVCA433, which has high endogenous levels of E-cadherin. We introduce human transgenes for Snail, Slug and Zeb1 by lentiviral infection and compare the resulting sublines by filtration. Similar to previous results in the SKOV3^EMT1/2^ cell lines, EMT-altered OVCA433 cells have increased deformability, as shown by the lower % retention of the modified cells compared to the non-modified control (Figs 1c, 2a).

Evaluating the % retention across the entire panel of EMT cells reveals that the mean % retention of all epithelial-like cells is significantly higher than the mesenchymal-like cells, indicating that epithelial-type cells are more resistant to deformation through micron-scale pores than those with mesenchymal phenotype (Fig. 2a, Supplementary Fig. 5a). By Western blot analysis, we confirm that across our panel of 10 different EMT related cell types, all samples with retention above 80% are epithelial-like cells and express (i) increased levels of E-Cadherin and/or (ii) decreased levels of vimentin compared to cells with mesenchymal phenotype that show below 65% retention (Fig. 2b,c). While epithelial and mesenchymal-type cells show distinct morphologies in culture, the size distributions of cells in suspension are similar, and there is a weak correlation of cell-to-pore size ratio with % retention, indicating that cell size does not significantly alter % retention (Supplementary Fig. 2c, correlation coefficient 0.14). Importantly we observe a single large peak in the cell size distributions, indicating that clustering of epithelial-type cells is not a major contributor to pore occlusion. Imaging of membranes after filtration confirms that 93–98% of occluded pores are occluded by single cells (Supplementary Fig. 6); these results also show that cells do not undergo lysis as they occlude pores. We cannot exclude that altered friction between the cell and wall may impact % retention, especially as cell deformability affects the normal force exerted on the cell, and thus softer cells may experience reduced friction^26^. Indeed, cells with increased metastatic potential show reduced surface friction during transit through pores^38^. However, we do not observe any increase in % retention when mesenchymal-type cells are filtered with BSA treatment that blocks cell-surface interactions (Supplementary Fig. 7). We also confirm there are no observable differences in non-specific binding across cell lines (Supplementary Table 1). In addition, we find a marked >3-fold reduction in % retention when epithelial-type OVCA433-GFP cells are treated with cytochalasin D (Supplementary Fig. 3); these results substantiate that mechanotype plays a major role in determining % retention.

To determine if PMF could inform the user of cell phenotype based on mechanotype alone, we conduct a blind screen across the EMT panel of cell lines, whose identities are not known to the user; these results show that PMF can be used to predict whether cells are more likely to be mesenchymal- or epithelial-like based on their mechanotype (Supplementary Fig. 5). We confirm the EMT phenotype of these samples by immunoblotting (Supplementary Fig. 8).

To gain insight into the molecular origins of the altered mechanotype in EMT, we perform immunofluorescence and confocal microscopy to investigate the organization of key structural components, such as actin, microtubules, and the nucleus, which are major contributors to mechanotype^8^,^18^,^39^,^40^. We focus our analysis on pairs of cells that exhibit marked differences in mechanotype: mesenchymal-type cells (MOSE HRAS^V12^, OVCA433 ZEB1) show an increased density of cortical actin compared to the control cells (MOSE pWZL blast, OVCA433-GFP) (Fig. 2e,f). There are neither observable differences in microtubule structure nor in cell nucleus morphology (Fig. 2e,g). We also investigate the nuclear-to-cytoplasmic size ratio, which could also impact cell filtration rates. While the reduced nuclear-to-cytoplasmic size ratio of the Zeb1-expressing cells could help to explain their reduced % retention, the nuclear-to-cytoplasmic size ratio is not significantly different between MOSE HRAS^V12^ and the control pWZL blast cells (Supplementary Fig. 9). Therefore, a reduced nuclear-to-cytoplasmic size ratio is not sufficient to explain the softer mechanotype we observe for all mesenchymal-type cells.

### Detecting the effects of drugs on cell mechanotype

**Figure 3:**
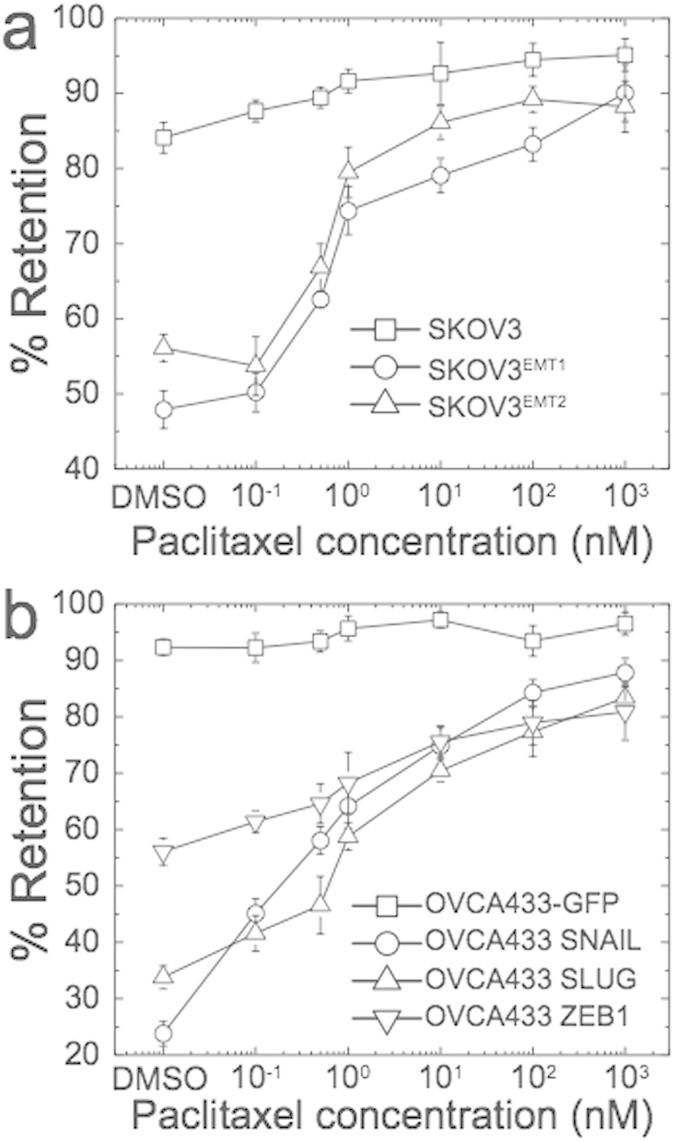
Effects of paclitaxel on the mechanotype of epithelial versus mesenchymal-type cells. Cells are treated with paclitaxel for 24 hrs prior to filtration through a 10 μm membrane. (**a**) Percentage retention of SKOV3 control and modified cells are measured after filtration at 2.1 kPa for 50 s. (**b**) Percentage retention of OVCA433-GFP control and modified cells are measured after filtration at 2.8 kPa applied for 40 s. Compared to the DMSO-treated control cells, there is an increase in % retention of 1 nM-treated cells: 17% ± 2%, p = 3.1 × 10^*−*3^ for SKOV3^EMT1^; 23% ± 6%, p = 1.9 × 10^*−*5^ for SKOV3^EMT2^; 15% ± 6%, p = 1.4 × 10^*−*2^ for OVCA433 SNAIL; 10% ± 6%, p = 1.2 × 10^*−*2^ for OVCA433 SLUG; 23% ± 2%, p = 8.9 × 10^*−*5^ for OVCA433 ZEB1. We note that with higher applied stresses, epithelial-type cells also exhibit a similar increase in retention (Supplementary Fig. 14). Each data point represents mean ± S.D.

A potentially valuable application of PMF would be to identify anti-cancer compounds by screening cells on the basis of mechanotype. An effective chemotherapy strategy is to impair cell division by targeting cytoskeletal components that are essential for proliferation, such as microtubules^41^,^42^. One common drug is paclitaxel, which is used in treatment of ovarian, breast, and non-small cell lung cancers. To validate the utility of PMF to detect differences in cell deformability following drug response, we treat SKOV3 and OVCA433 cells with paclitaxel at 0.1 to 1000 nM; the IC_50_ for these cells is within this range^43^. We use a pressure of 2.1 kPa, which results in ~45 to 65% retention for the mesenchymal-type cells prior to drug treatment, ensuring a dynamic range for detecting softer or stiffer cells when multiple samples are screened in parallel. Over the range of paclitaxel concentrations, we observe that increasing doses of paclitaxel cause a significant >30% increase in retention (Fig. 3). Even for a physiologically relevant dose of 1 nM^43^, there is a statistically significant increase in retention for mesenchymal-type cells compared to DMSO-treated control cells. In contrast, epithelial-like cells exhibit a smaller <10% increase in retention. For all measurements, we verify that cell viability remains over 94% (average viability 99%, Supplementary Table 2). We also confirm that below 1 nM paclitaxel, where we observe the largest increase in % retention compared to the control-treated sample, there are no significant differences in cell size (Supplementary Fig. 2).

### Mechanotyping cisplatin-sensitive versus -resistant human ovarian cancer cells

To explore EMT in a context relevant to ovarian cancer treatment, we investigate the mechanotype of drug-resistant versus -sensitive ovarian cancer cells. A major challenge in treating human ovarian cancer is to distinguish cells that develop resistance to common chemotherapy drugs, such as cisplatin, which frequently result in recurrence and poor patient outcome^44^,^45^. If we could apply PMF to detect ovarian cancer cells that are sensitive to cisplatin (SKOV3, OVCAR5) and their drug-resistant counterparts (SKOV3-CisR, OVCAR5-CisR) using cell mechanotype as a biomarker, our assay could have potential application in clinical settings.

**Figure 4:**
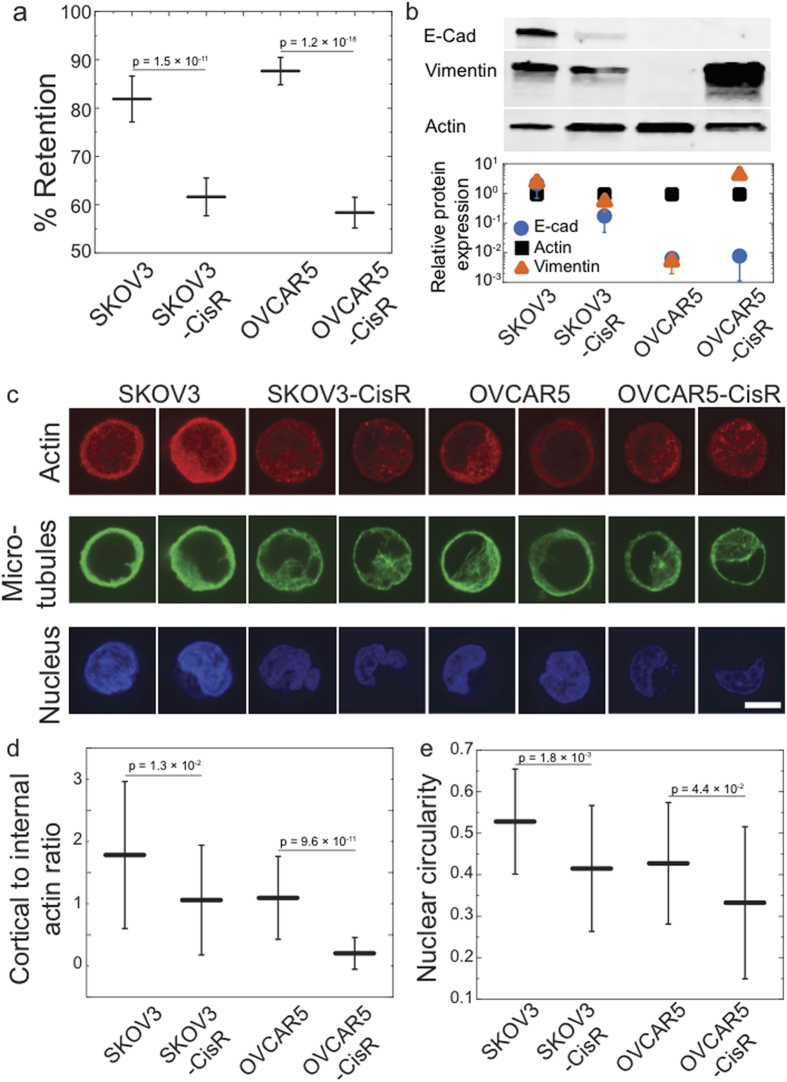
Cisplatin-sensitive and -resistant human ovarian cancer cells show altered mechanotype. (**a**) Percentage retention of cisplatin sensitive cells, SKOV3 and OVCAR5, versus cisplatin resistant (CisR) cells, SKOV3-CisR and OVCAR5-CisR, after filtration with 10 μm pore membrane at 2.1 kPa for 50 s. Each data point represents mean ± S.D. (**b**) Western blot analysis and quantification of protein biomarkers for epithelial and mesenchymal-type cells, E-cadherin (ECad) and vimentin protein levels normalized to the actin loading control. Data points represent mean ± S.D. (**c**) Confocal images of immunolabeled cells. Nuclei are stained with DRAQ5. Two representative images are shown for each cell type. Scale, 10 μm. (**d**) Cortical to internal actin ratio. (**e**) Nuclear circularity.

We generate drug resistant cells by treatment with cisplatin over 12 months^23^. These cells show similar size compared to their drug-sensitive counterparts (Supplementary Fig. 2). PMF reveals that both SKOV3-CisR and OVCAR5-CisR samples have lower % retention compared to the cisplatin sensitive cells (Fig. 4a), indicating that the drug-resistant cells are more deformable than their drug-sensitive counterparts. Since the EMT status of these cells had not previously been determined, we perform immunoblotting to verify if these softer, CisR cells also have biochemical markers that characterize mesenchymal-type cells. The SKOV3-CisR cells show reduced levels of E-Cadherin, a common feature of mesenchymal-type cells (Fig. 4b). While OVCAR5 cells inherently have low levels of E-Cadherin, the OVCAR5-CisR cells show a marked increase in vimentin levels; this is also a hallmark of cells with mesenchymal phenotype.

To explore the origins of the altered mechanotype in the cisplatin sensitive and resistant cells, we investigate the structure of actin, microtubules, and the nucleus by immunofluorescence and confocal imaging. While microtubules exhibit similar structure in both cell types, the cisplatin sensitive cells have a denser cortical actin network compared to CisR cells (Fig. 4c,d). We also observe differences in nuclear morphology: all cells exhibit nuclei with a variety of morphologies including ovoid and kidney-shapes, but the circularity index is reduced for CisR cells, reflecting that their nuclei exhibit more irregular shapes than the cisplatin sensitive cells (Fig. 4c,e). Altered physical properties of the nucleus may also contribute to the altered filtration behavior of the CisR cells, as the nucleus rate-limits the transit of cells through micron-scale pores^18^.

## Discussion

Here we show how PMF enables comparative measurements of cell mechanotype simultaneously across multiple cell samples. Using PMF, we observe a strong correlation between biochemical and mechanical phenotypes of cells through EMT, including when induced by acquisition of drug resistance, as well as by overexpression of specific master regulators that are implicated in EMT. We find that the mesenchymal phenotype following EMT, which is associated with cancer progression and drug resistance, results in increased deformability across the cell panel. These findings were made possible by the ability of PMF to screen a panel of cells simultaneously. Moreover, the PMF method is scalable and significantly reduces the time required to analyze individual samples of cells; parallel measurements of cell deformability are not possible using existing methods.

Our results show a marked difference in the mechanotype of epithelial versus mesenchymal-type cells. Studies using conventional techniques have indicated a difference in the mechanotype of these cell types^22^,^23^,^31^,^32^. While we observe that mesenchymal-type cells have a softer mechanotype, previous studies revealed that mesenchymal-type cells are stiffer. For example, normal murine mammary gland (NMuMG) cells that are induced through EMT by TGF-beta-1 are more resistant to deformation^31^,^32^. Similarly, CisR ovarian cancer cells have a higher elastic modulus compared to the drug-sensitive controls^22^,^23^. However, those results are obtained for cells adhered to glass substrates (*E* ~ 70 GPa), which tend to have well-developed actin stress fibers and increased intracellular tension^23^. By contrast, PMF probes cells in a suspended state, where CisR cells have a lower density of cortical actin compared to the cisplatin sensitive cells. The mode of deformation between PMF and other techniques such as AFM is also distinct: PMF measures the ability of whole cells to deform through micron-scale pores that are ~40-70% of their size, whereas AFM indents cells over nm to sub-μm length scales.

While PMF robustly identifies epithelial- versus mesenchymal-type cells, the molecular origins of cell mechanotype remain to be fully understood. Dramatic alterations in cytoskeletal architecture occur with EMT. For example, cortical actin reorganizes to form stress fibers and structures that enable directional motility^31^; these modified actin structures can contribute to altered cancer cell mechanotype^4^,^46^. In the mesenchymal-like cisplatin resistant versus sensitive cells, the observed decrease in cortical actin provides a plausible mechanism for their softer mechanotype: levels of cortical actin regulate % retention, as observed in HL-60 and OVCA433-GFP cells treated with cytochalasin D or colchicine to induce either a decrease or increase in F-actin (Supplementary Fig. 3). However, in other types of mesenchymal-like cells, we observe an *increase* in cortical actin compared to their epithelial counterparts; this typically causes an increase in cell elastic modulus, and thus cannot fully explain the observed softer mechanotype of all mesenchymal-type cells. The CisR cells also exhibit irregular nuclear structure. Since we cannot dissect the individual contributions of cortical actin and the nucleus, altered % retention could also be caused by altered structure of the cell nucleus, which is a major contributor to cell mechanotype and the ability of cells to transit and migrate through narrow pores^18^,^47^. Numerous other mechanisms could impact cell mechanotype. For example, several actin-associated proteins are downregulated by Snail, such as CAPG capping protein and gelsolin^36^. Rho-mediated actin remodeling^22^ and nuclear lamins^18^ can also alter cell mechanical properties. Other factors such as myosin II regulate contractility and motility^48^; the transcription factors YAP1 and TAZ are also implicated in cell mechanotype^49^. PMF should enable further studies to elucidate the molecular mechanisms of mechanotype, for example by screening cells treated with siRNA or CRISPR libraries.

Our label-free mechanotyping assay also has potential for clinical applications, for example, to screen established cell lines and patient samples for prognosis and drug screening. This robust and high throughput method allows rapid analysis of ~10^5^–10^6^ cells per sample, which is important due to the cellular heterogeneity within a tumor. While we have investigated here cell samples with relatively uniform cell-to-pore size ratios, PMF screening using a range of pore sizes could enable screening clinical samples that may contain mixed populations of cells with different size distributions. PMF could thus complement and enhance existing methods for evaluating cancer cells and their response to drugs. Considering that key steps in metastasis require large deformations of cells, such as intravasation and extravasation, this simple assay could provide physiologically relevant insights into the behavior of cancer cells. More broadly, PMF could be valuable for identifying compounds that are relevant to other disorders that are characterized by altered cell mechanotype, from malaria to diabetes.

## Methods

### Cell culture

Human promyelocytic leukemia (HL-60) cells are cultured (5% CO_2_, 37 ^o^C) in RPMI-1640 media with L-Glutamine (Invitrogen) supplemented with 10% fetal bovine serum (FBS) and 1% penicillin-streptomycin (PenStrep, Gemini BioProducts, Calabasas, USA). To avoid spontaneous differentiation that can arise due to increased HL-60 cell density, we maintain these cells at a density below ~8 × 10^5^ cells/ml. Neutrophil-type cells (dHL-60) are generated by treating HL-60 cells with 5 μM all-trans retinoic acid (ATRA) (1 mM stock in ethanol, Sigma-Aldrich) for 5 days. Human ovarian cancer (SKOV3 and OVCA433) cells are cultured in DMEM (+L-Glutamine, +Glucose, +Sodium Pyruvate) supplemented with 10% FBS, 1% Anti-anti (Gibco), and 2.5 μg/ml Plasmocin Prophylactic (Invivogen). For OVCA433 cells and derivatives (Snail, Slug, Zeb1) as well as MOSE pWZL blast, we use the same media with the addition of blasticidin S HCl (5 μg/ml, Corning Cellgro); for the MOSE HRAS^V12^ cells, we add hygromycin (4 μl/ml, Corning Cellgro); and for the MOSE CCNE1 cells, puromycin (5 μg/ml, Corning Cellgro). To culture the pairs of cisplatin-sensitive and -resistant cells, SKOV3/SKOV3-CisR and OVCAR5/OVCAR5-CisR, we use DMEM with 10% FBS, 1% Penicilin-Streptomycin, and 10 μM cisplatin (Sigma-Aldrich) for the resistant cells. Adherent cells are harvested by washing with 1× Phosphate-Buffered Saline (PBS, DNase-, RNase- & Protease- free, Mediatech, Manassas, USA), treating with trypsin, and resuspending in fresh medium. To minimize clusters of cells, cell suspensions are passed through a 35 μm filter (BD Falcon) prior to each measurement. The identity of each cell line was confirmed by short tandem repeats (STR) profiling (Laragen Inc).

### EMT Transformation

To generate EMT gene expression clones, *SNAI1*, *SNAI2*, and *ZEB1* cDNA (Open Biosystems) are individually inserted into the pLenti6.3/V5-DEST vector (Invitrogen; *ZEB1* and *SNAI2*) or pLenti4/V5-DEST vector (Invitrogen; *SNAI1*) by the Gateway cloning system (Invitrogen). OVCA433 cells are transduced with lentiviral expression plasmids harboring *SNAI1*, *SNAI2*, and *ZEB1* or GFP (control) and selected with 5 μg/mL blasticidin (InvivoGen) for 6 d prior to use. SKOV3^EMT^ cells are established by chronic exposure to a combination of 0.2 μM PD0332991 and 0.2 μM SNS032 for over 12 months (Taylor-Harding *et al* 2014, Submitted)^50^. SKOV3-CisR and OVCAR5-CisR cells are generated by culturing with 10 μM cisplatin over 12 months^23^.

### Drug treatment

A stock solution of paclitaxel (25-950-CQC, Corning Cellgro) is prepared in DMSO to 1 mM. Cells are treated with the desired concentration of drug for 24 hrs prior to measurements.

### Parallel microfiltration

The PMF device is assembled using polycarbonate membranes (Isopore, Millipore) of 5, 8, and/or 10 μm pore diameter. Wells are loaded with 1% w/w bovine serum albumin (BSA) solution (Fisher) for 1 hr at 37 ^o^C, and then emptied and air dried at least 1 hr before each experiment. Cell suspension at a concentration of 0.5 × 10^6^ cells/ml (ovarian cancer cells) and 1.0 × 10^6^ cells/ml (HL-60 cells) is loaded into each well. Well-defined air pressures from 0.7 to 7 kPa are applied using a custom-built manometer (Supplementary Fig. 10) and monitored using a pressure gauge (Noshok Inc., Berea, OH, USA). We determine % retention by collecting the sample suspension remaining in the top well and measuring the mass using a precision balance (Northeast Scale Inc., Hookset, NH, USA). To measure cell number and size distributions, we use an automated cell counter (TC20, BioRad). We also verify that cells in suspension do not cluster over our experimental time scale of ~15 minutes (Supplementary Fig. 11).

### Immunofluorescence staining and image analysis

Cell suspensions are placed in chambers of Millicell EZ slides (Millipore) for ~1.5 hours at 37 ^o^C before fixation with paraformaldehyde (4% in PBS) for 20 minutes at room temperature. Cells are then permeabilized with 0.2% Triton X-100 for 10 minutes and blocked with 3% BSA in PBS for 2 hours. To stain cytoskeletal components, primary and secondary antibodies (Sigma-Aldrich) are sequentially applied to cells for 1 hour: Anti-Actin (CAT#: A2066); Anti-Rabbit IgG F(ab′)2 fragment - Atto 488 (CAT#: 36098; λ_ex_−500 nm; λ_em_−522 nm); Monoclonal Anti-α-Tubulin (CAT#: T5168); Anti-Mouse IgG−Atto 550 (CAT#: 43394; λ_ex_-550 nm; λ_em_-576 nm). To label the cell nucleus, we use the DNA-intercalating dye, DRAQ5 (Fisher Scientific). Imaging is performed using a confocal microscope (LSM 5 EXCITER, Laser Scanning Microscope, Zeiss) equipped with a 63× objective (63x/1.2 W Korr UV-VIS-IR, C-APOCHROMAT, Zeiss). To measure actin signal, an Argon laser (488 nm) and BP505-530 filter are used; to measure microtubule signal, a Helium-Neon laser (543 nm) and BP560-615 filter are used; to measure nuclear signal, a Helium-Neon laser (633 nm) and LP650 filter are used. Midplane confocal images are analyzed using ImageJ. For analysis of cortical-to-internal actin ratio, we measure the integrated signal of immunostained actin in the whole cell and an internal region that is 1 μm from the boundary of the cell; actin intensity in the cortical region is determined by subtracting the intensity of the internal region from the total actin intensity in the whole cell. Circularity is calculated as 4πA/P^2^, where A and P are the area and perimeter of individual nuclei; for a perfect circle, the circularity value is one.

### Western blots

Whole cell protein extracts are separated by SDS-PAGE and transferred onto PVDF membranes using semi-dry transfer (BioRad). After incubation with 5% milk in PBST (10 nM Tris, pH 7.4, 150 nM NaCl, 0.1% Tween 20) for one hour, the membrane is probed with antibodies against proteins of interest including E-cadherin (CAT#: 610181, BD Biosciences), vimentin (CAT#: MS-129-PO, Thermo Scientific), and actin (CAT#: MA5-15739, Thermo Scientific) as a loading control. Antibodies are incubated at 4 °C for 12 h followed by three washes of 10 minutes each in PBST. Proteins are then detected with fluorescent-conjugated antibodies, Goat anti-Mouse IgG IRDye 680LT (CAT#: 827-11080) and Goat anti-Rabbit IgG IRDye 680RD (CAT#: 926–68170). Immunoblots are visualized using a Li-Cor Odyssey Infra-red imaging system.

### Soft agar assay

Anchorage-independent growth of MOSE epithelial and mesenchymal-type cells is tested by colony formation in soft agar. We plate 1-ml of DMEM (+L-Glutamine, +Glucose, +Sodium Pyruvate) supplemented with 10% FBS and 1% Anti-anti (Gibco) containing 1.2% agar into each well of a 6-well plate; ~10^4^ cells are then suspended in 1% agarose containing culture medium. After 2–3 weeks, colonies are stained with 0.5 mg/ml iodonitrotetrazolium chloride and the number of colonies is determined across triplicate wells for each cell line.

### Statistical methods

We perform PMF using 4 wells for a single sample in parallel multiple sample measurements. All data is obtained from at least 3 independent measurements and is expressed as mean ± S.D. We use the Student’s t-test method to analyze the results and to obtain p-values.

### Numerical modeling

The flow rate is proportional to the number of available pores, *Q(t)* *=* *qn(t)*, where *q* is the flow rate through a single pore and *n(t)* is the number of open pores at time, *t*. While *q* is set by the applied pressure and pore dimensions and thus remains constant throughout a single experiment, the total flow rate, *Q(t)*, changes over time as it is determined by the number of available pores. We model the filtration process using successive iterations over time: in a given time interval, *τ*, each available pore can be encountered by a single cell. To model filtration for a given set of conditions, we determine *τ* by *(qc)*^*−1*^, which is the time for the fluid volume per cell, *1/c*, to pass through a single pore. Here, *c* is the density of the cell suspension, which remains relatively constant before and after the filtration measurement (Supplementary Fig. 12). In a given time window, ∆*t*, the total number of iterations is *J* = ∆*t/ τ.* We use the least squares method to fit our experimental results, where the only adjustable parameter is *χ*, the fraction of cells that occlude the filtration pores. Other parameters in the model are known: the initial number of pores in the filtration area is obtained by measuring the membrane pore density and total filtration area in a single well (Supplementary Fig. 13); the medium flow rate through single pores is calculated using Poiseuille’s law and is in agreement with the experimentally measured values. By considering the fraction of cells that occlude the pores within each time interval, we can recapitulate the decreasing permeability of the membrane: as filtration proceeds, an increasing volume of cell suspension and thus, number of cells, arrive at the pores; the fraction of occluded pores increases; the total flow rate subsequently decreases; and progressively fewer cells are brought to the membrane. A similar time-dependent increase in fluidic resistance is considered in crossflow membrane filtration of colloidal suspensions^51^,^52^.

## Additional Information

**How to cite this article**: Qi, D. *et al.* Screening cell mechanotype by parallel microfiltration. *Sci. Rep.* **5**, 17595; doi: 10.1038/srep17595 (2015).

## Supplementary Material

Supplementary Information
