# Supplementary material for: Screening cell mechanotype by parallel microfiltration
Source: Sci Rep. 2015 Dec 2;5:17595. doi: 10.1038/srep17595 (PMC4667223; doi:10.1038/srep17595)
Supplement: Supplementary Information [file srep17595-s1.pdf]

Supplementary Information for

Screening cell mechanotype by parallel microfiltration

Dongping Qi, Navjot Kaur Gill, Chintda Santiskulvong, Joshua Sifuentes, Oliver Dorigo, JianYu Rao, Barbie Taylor-Harding, W. Ruprecht Wiedemeyer, Amy C. Rowat*

*Corresponding author. E-mail: rowat@ucla.edu.

Supplementary Notes

1. Parallel filtration device and measurement. To obtain a pressure-tight seal between the pressure chamber and the top plate, we place a silicone seal (McMaster Carr, USA) in between the top plate and the pressure chamber. Two aluminum plates affixed to the top and bottom of the 'sandwich' are used to clamp the entire setup together (**Fig. 1**). To facilitate flow of cell suspensions through the porous membrane, we drill 1 mm holes into the bottom of each well of the bottom plate (**Supplementary Fig. 15**). Cross-sample contamination and leakage across individual sample wells are prevented by sealing each well with an O-ring; a silicone mat punctuated with holes that correspond to the array of wells can alternatively be used to isolate the contents of individual wells.

2. Protocol for PMF assay. (1) Place silicone mat on bottom plate, or O-rings in each well of the bottom plate (**Fig. 1**). (2) Position porous membranes on top of the bottom plate and sealant. (3) Place the top plate on the membrane/O-rings, using the steel bolts at the edge of the plates to guide the alignment of the top and bottom wells (**Supplementary Fig. 15**); thereafter clamp both plates using the edge steel bolts. (4) To minimize cell-device surface interactions, pretreat the device by placing 800 μl of fresh bovine serum albumin (BSA) solution (1% w/w in de-ionized water, filtered through 0.2 μm Surfactant-Free Cellulose Acetate (SFCA) membrane filter unit (Nalgene, Thermo Scientific) in each top well before use. Incubate the setup at 37°C for one hour. Thereafter, remove the BSA solution from the wells and air dry the

device. (5) Place the parallel filtration device in a shallow ~ 7 mm water bath; this prevents sample drainage due to gravity. (6) Measure the density of the cell suspension using a particle/cell analyzer (e.g. BioRad TC20 Cell Counter or Coulter Counter) and then place 750 µl of cell suspension with a concentration of 10^6 cells/ml into each well. (7) Position the pressure chamber and tighten the entire device together to achieve an airtight seal. (8) Apply a well-defined pressure for 20 – 50 s. (9) Remove the pressure chamber and collect the samples retained in the top wells for measurement of retained volume and/or cell number.

3. Pore size dependence of filtration. To determine the pore size dependence of filtration, we use three types of polycarbonate membranes (Isopore, Millipore, USA) with pore sizes of 5, 8, and 10 µm. Since membrane porosity is essential for filtration, we characterize the membranes using confocal microscopy, taking advantage of the zero-reflection of the hollow area of pores. We observe that some pores are connected together, and define these as pore clusters (**Supplementary Fig. 14**). To obtain the porosity for each membrane, we perform quantitative image analysis using ImageJ to determine the ratio of pore or cluster area to total membrane area, as shown in **Supplementary Table 3**.

Supplementary Figures

Supplementary Figure 1

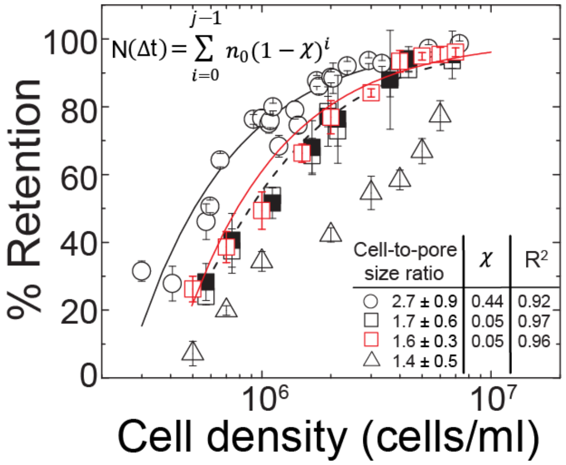

Supplementary Figure 1. Quantification of cell filtration by % retention. Filled squares represent cell number % retention; open symbols show fluid mass % retention; both parameters can be used equivalently to quantify filtration.

Supplementary Figure 2

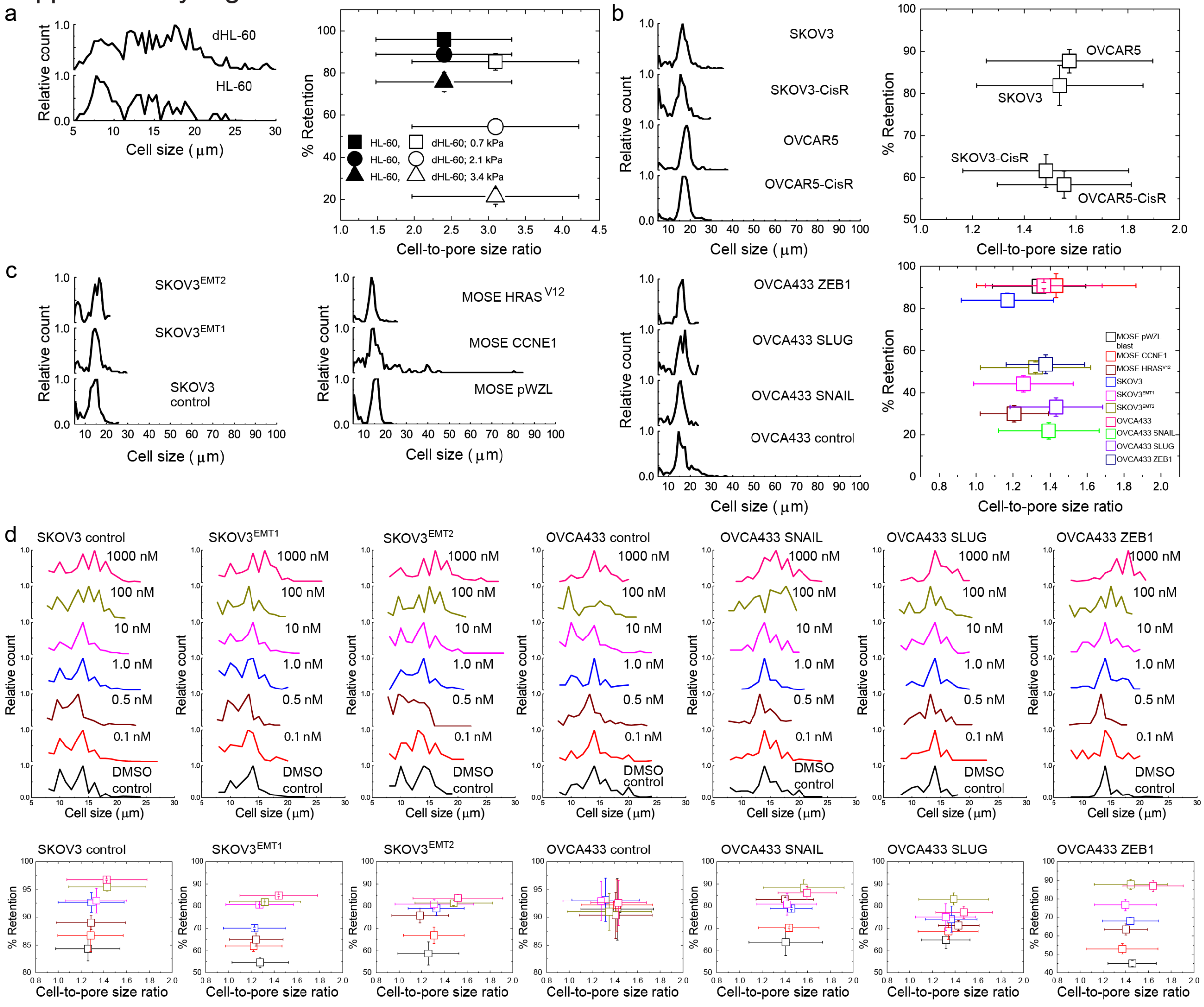

Supplementary Figure 2. Size distribution and filtration of cells. Percentage retention versus cell-to-pore size ratios for: **(a)** HL-60 and dHL-60 cells; **(b)** cisplatin-sensitive (CisS) and cisplatin-resistant (CisR) cells; **(c)** epithelial and mesenchymal-type cells in the cell panel; and **(d)** cells treated with paclitaxel and carrier DMSO control.

Supplementary Figure 3

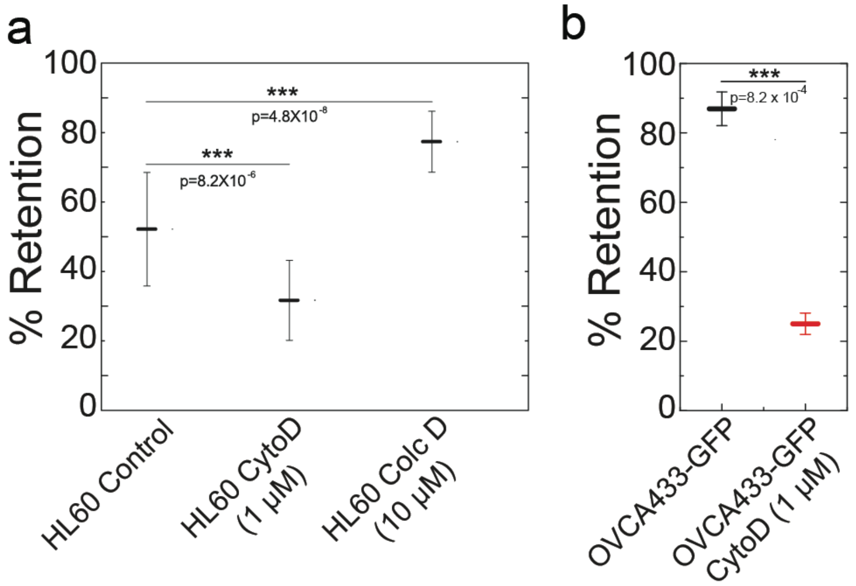

Supplementary Figure 3. Effect of F-actin on filtration. (a) HL-60 cells treated with cytochalasin D (Cyto D) or colchicine (Colc) to induce either a decrease or increase in F-actin. Measurement conditions: 8 μm pore membrane; 0.7 kPa applied for 20 s. (b) OVCA433-GFP cells treated with Cyto D. Mean (Horizontal line) ± S.D. (vertical line). P-values reflect statistical significance determined by Student's t-test.

Supplementary Figure 4

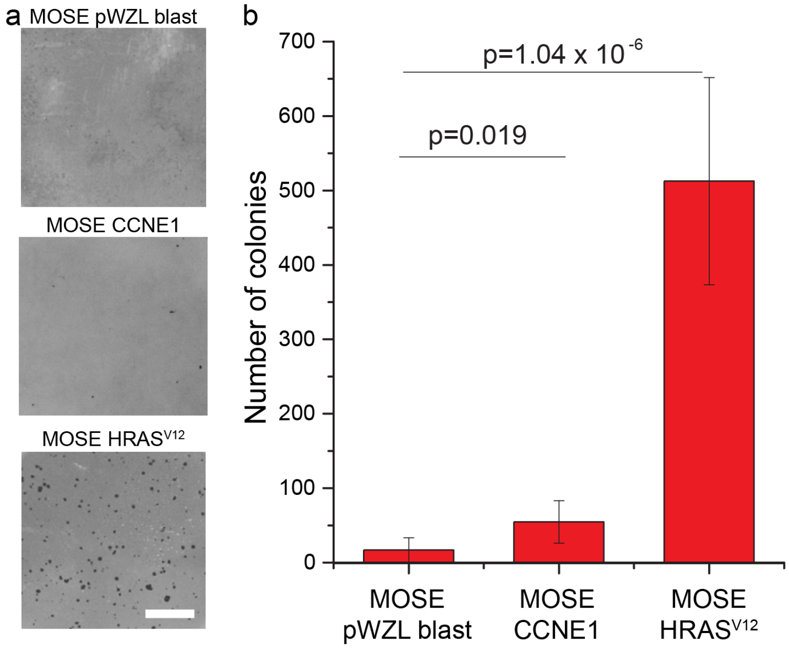

Supplementary Figure 4. Mesenchymal-type cells form colonies in soft agar assay. MOSE control (pWZL blast) cells, MOSE CCNE1, and MOSE HRASV^{12} cells are embedded in soft agar. **(a)** Images of cell colonies labeled with 0.5 mg/ml iodonitrotetrazolium chloride after 21 days. Brightness and contrast of the grayscale images is adjusted (to 122 brightness; 40 contrast) for visualization of MOSE pWZL blast and MOSE HRASV^{12} samples. Scale, 5 mm. **(b)** Quantitative analysis of colony number reveals that MOSE HRAS^{V12} cells form a greater number of colonies compared to the CCNE1-modified and non-modified control cells. Bar graphs show mean values obtained over 2 independent experiments; vertical lines denote ± S.D.

Supplementary Figure 5

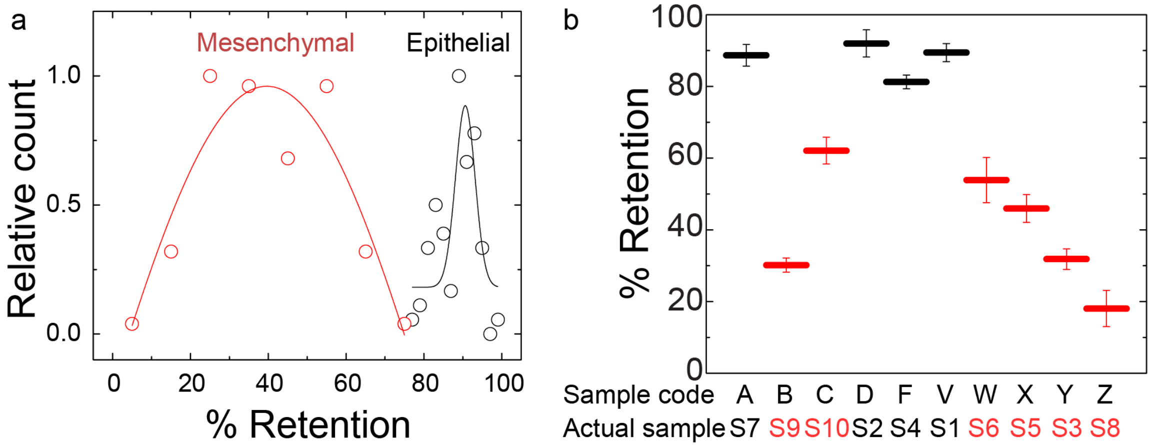

Supplementary Figure 5. Predicting epithelial and mesenchymal-type cells across the EMT cell panel. **(a)** Probability distributions of % retention for cells across the EMT cell panel. Raw data is obtained from panel reference measurements, as shown in Fig. 2a, and fitting is performed using a single peak Gaussian function. Peak center positions are 39.6 ± 46.1 for mesenchymal cells and 90.6 ± 2.5 for epithelial cells. **(b)** Blind mechanotyping assay performed with the same panel of cell lines using the same measurement conditions as in Fig**. 2a.**

Supplementary Figure 6

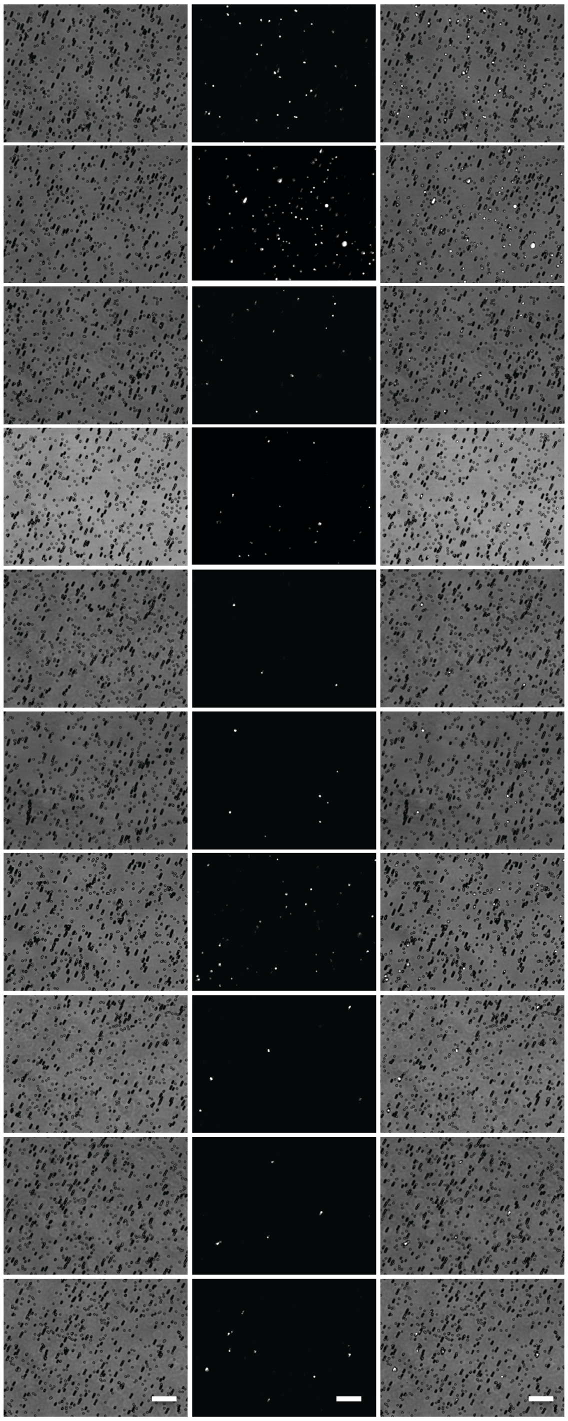

	% Pores occluded	% Occluded pores occluded by single cells	% Retention
MOSE pWZL Blast	5.4 ± 1.4	95.6 ± 0.1	90.3 ± 0.1
MOSE CCNE1	9.3 ± 1.3	93.0 ± 0.2	92.7 ± 0.2
MOSE $HRAS^{V12}$	1.3 ± 1.0	97.7 ± 0.0	34.9 ± 0.0
SKOV3	7.5 ± 2.5	95.5 ± 0.1	81.0 ± 0.1
$SKOV3^{EMT1}$	1.0 ± 0.5	93.9 ± 0.0	47.4 ± 0.0
$SKOV3^{EMT2}$	2.0 ± 1.4	93.0 ± 0.1	54.6 ± 0.1
OVCA433-GFP	6.4 ± 2.3	97.6 ± 0.0	88.6 ± 0.0
OVCA433 SNAIL	1.0 ± 0.6	97.0 ± 0.0	21.7 ± 0.0
OVCA433 SLUG	1.2 ± 0.4	94.9 ± 0.0	29.5 ± 0.0
OVCA433 ZEB1	1.8 ± 0.6	93.7 ± 0.0	60.8 ± 0.0

Supplementary Figure 6. Images of membranes after filtration. Fluorescence images reveal cells that are labeled with Cell Tracker dye. Percentage of pores that are occluded by single cells/ cell clusters that are larger than 18 μm are displayed in the right column. Scale, 100 μm.

Supplementary Figure 7

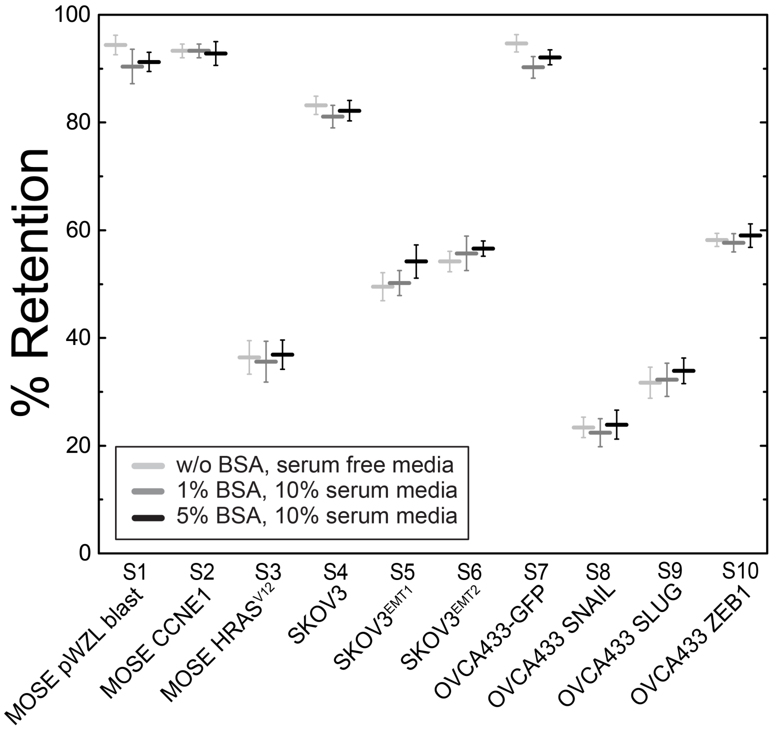

Supplementary Figure 7. Effects of BSA in % retention. Filtration of the panel of ovarian cancer cells is performed with varying concentrations of bovine serum albumin (BSA).

Supplementary Figure 8

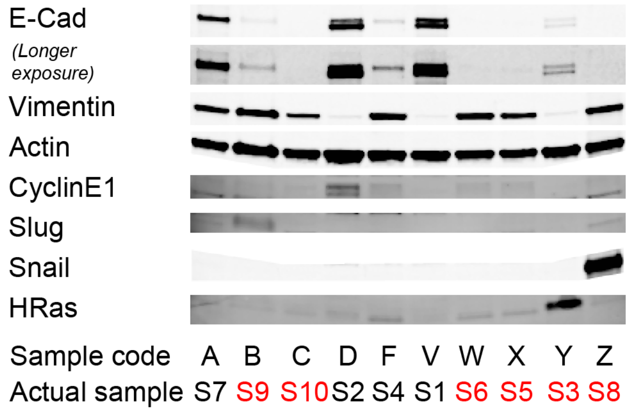

Supplementary Figure 8. Western blot confirmation of epithelial and mesenchymal-type samples run in the blind assay. Black denotes epithelial-type cell samples, while samples of mesenchymal-type cells are shown in red.

Supplementary Figure 9

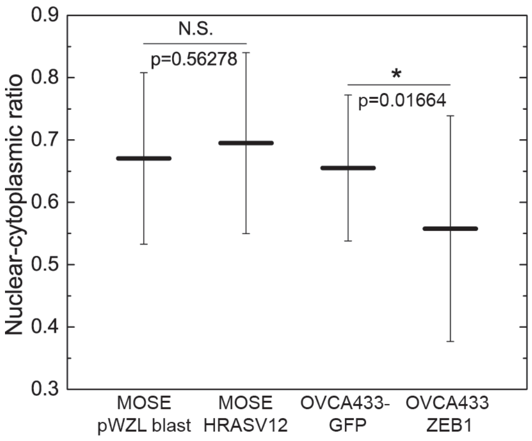

Supplementary Figure 9. Nuclear-to-cytoplasmic ratio of representative epithelial and mesenchymal-type cell lines.

Supplementary Figure 10

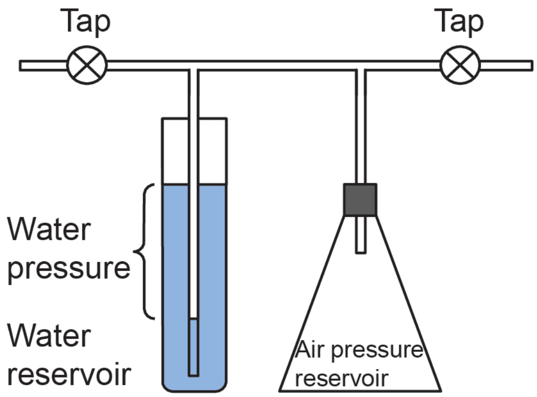

Supplementary Figure 10. Schematic illustration of a manometer. To build desired pressure (0-7 kPa) in the air pressure reservoir: close right tap, open left tap, blow in air from the air source (for example, manometer), and then close left tap. The pressure is indicated as the water pressure height. After a well-defined pressure is established, the right tap is opened to introduce air pressure to the pressure chamber of the parallel microfiltration platform.

Supplementary Figure 11

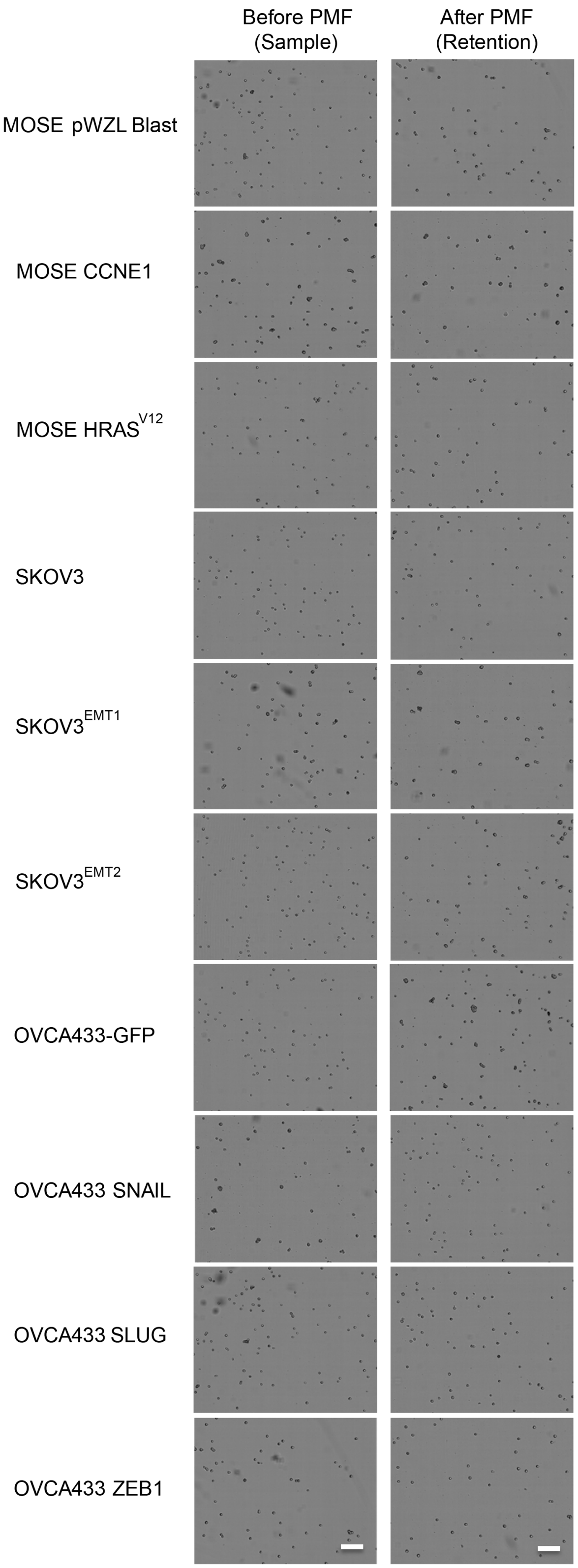

Supplementary Figure 11. Images of cell suspensions before and after PMF. Scale, 100 μm.

Supplementary Figure 12

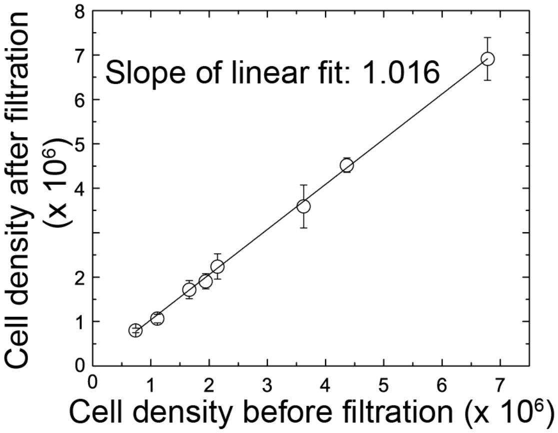

Supplementary Figure 12. Cell density before and after filtration. HL-60 cells are filtered through 8 μm pore membranes at 0.7 kPa for 20 s. After filtration, the cell density is measured from samples retained in top wells. Each data point represents mean ± S.D. Solid line represents linear fit to the data.

Supplementary Figure 13

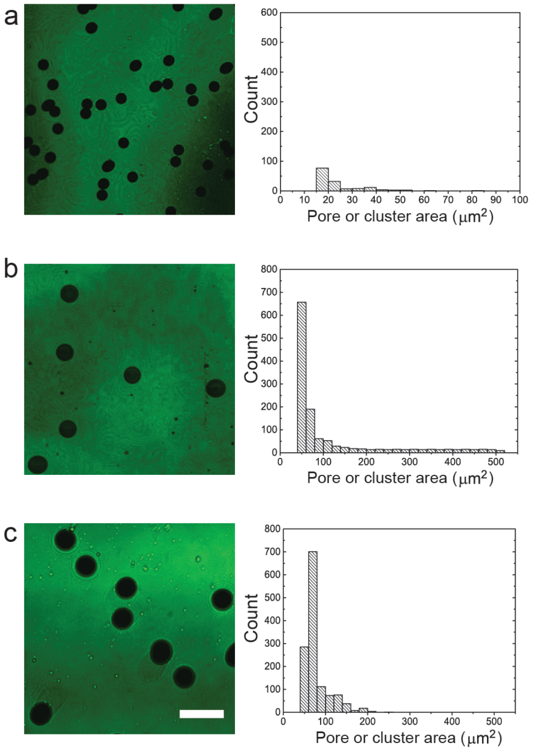

Supplementary Figure 13. Characterization of the porous membranes. (**a-c**) Representative images of porous membranes with 5, 8, and 10 μm pores. For each membrane, five confocal images are analyzed using ImageJ to obtain the pore (/cluster) histograms. We define clusters as any porous region that has an area greater than 1.5 pores. Further quantification of membrane porosity is provided in **Supplementary Table 3.** Scale bar, 20 μm.

Supplementary Figure 14

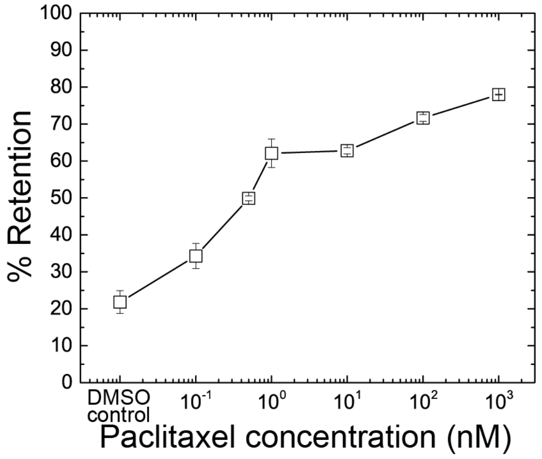

Supplementary Figure 14. Effects of paclitaxel on the filtration behavior of epithelial-like cells. SKOV3 cells are drug-treated 24 hrs prior to being filtered through 10 μm pore membranes at 2.8 kPa for 50 s. Each data point represents mean ± S.D.

Supplementary Figure 15

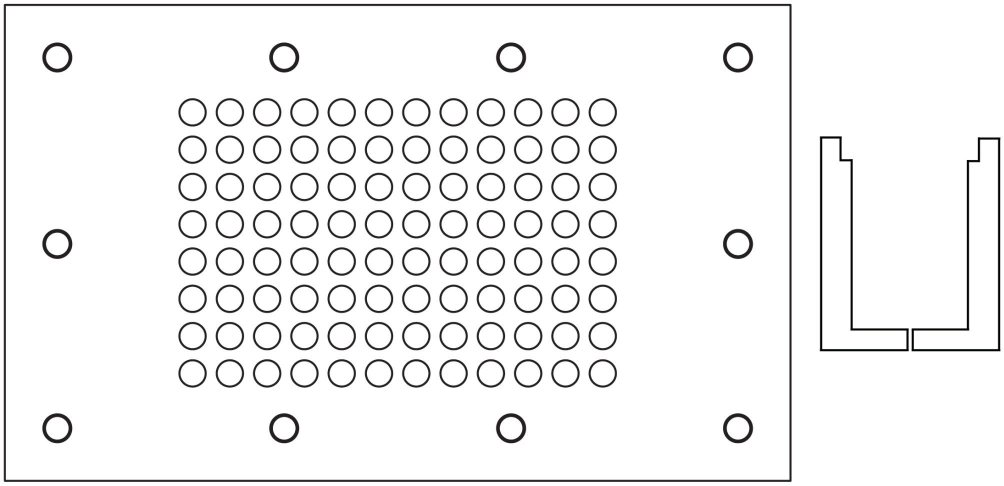

Supplementary Figure 15. Schematic illustration showing design of the 96-well plate. Left: top view of the 96-well plate. Thick circles around the perimeter of the plate represent holes for PMF assembly using steel bolts. Thin circles represent loading wells that each has a diameter of 0.64 cm (¼″) and depth of 2.54 cm (1″). Right: side view of a single well of the bottom plate. The bevelled structure at the top of the well is designed to hold an O-ring. Alternatively, a PDMS sealing mat can be used to achieve an air-tight seal. The bottom 1 mm hole facilitates filtration. Plates are machined out of Perspex (poly(methyl methacrylate) or PMMA). Drawing is not to scale.

Supplementary Tables

Supplementary Table 1. Investigating cell-surface interactions in % retention. Quantification of the number of cells recovered from wells after a filtration experimentation of ~15 min. Cell suspensions were then collected and cell densities were measured. Observed error for cell density measurements is ± 4 x 10^4.

Sample	Cell density of sample before PMF	Cell density of retained fluid after PMF
**MOSE pWZL Blast**	4.6×10^5	4.0×10^5
**MOSE CCNE1**	6.0×10^5	5.9×10^5
**MOSE $HRAS^{v12}$**	4.2×10^5	4.2×10^5
**SKOV3**	5.6×10^5	5.3×10^5
**$SKOV3^{EMT1}$**	5.9×10^5	5.4×10^5
**$SKOV3^{EMT2}$**	5.7×10^5	5.6×10^5
**OVCA433-GFP**	5.1×10^5	5.0×10^5
**OVCA433 SNAIL**	5.2×10^5	5.0×10^5
**OVCA433 SLUG**	4.7×10^5	4.2×10^5
**OVCA433 ZEB1**	5.1×10^5	5.2×10^5

Supplementary Table 2. Effect of paclitaxel (TAX) treatment on cell viability. Observed error for cell viability measurements is ± 2%.

Sample/ Conditions	SKOV3	SKOV3^{EMT1}	SKOV3^{EMT2}	OVCA433-GFP	OVCA433 SNAIL	OVCA433 SLUG	OVCA433 ZEB1
No treatment	98%	98%	100%	98%	99%	99%	97%
0.1% DMSO (control)	98%	98%	96%	95%	97%	97%	96%
0.1 nM TAX	98%	98%	98%	94%	98%	95%	98%
0.5 nM TAX	99%	99%	99%	94%	99%	99%	99%
1 nM TAX	96%	96%	98%	95%	99%	99%	96%
10 nM TAX	97%	96%	96%	95%	98%	98%	98%
100 nM TAX	98%	96%	96%	96%	96%	99%	98%
1000 nM TAX	96%	98%	95%	95%	98%	100%	97%

Supplementary Table 3. Characterization of membrane porosity.

Membrane	Total porosity (%) of single pores	Total porosity (%) of pore clusters	Total porosity (%)
5 µm	6.8 ± 0.7	2.5 ± 1.4	9.3 ± 2.0
8 µm	4.0 ± 0.3	0.7 ± 0.1	4.8 ± 0.4
10 µm	6.0 ± 0.3	1.4 ± 0.2	7.3 ± 0.4
